# Upwelling intensity and source water properties drive high interannual variability of corrosive events in the California Current

**DOI:** 10.1038/s41598-023-39691-5

**Published:** 2023-08-10

**Authors:** Julia Cheresh, Kristy J. Kroeker, Jerome Fiechter

**Affiliations:** 1 Department of Ocean Sciences, University of California, Santa Cruz, 1156 High Street, Santa Cruz, CA 95064 USA; 2 Department of Ecology & Evolutionary Biology, University of California, Santa Cruz, Santa Cruz, 95060 USA

**Keywords:** Marine chemistry, Physical oceanography

## Abstract

Ocean acidification is progressing rapidly in the California Current System (CCS), a region already susceptible to reduced aragonite saturation state due to seasonal coastal upwelling. Results from a high-resolution (~ 3 km), coupled physical-biogeochemical model highlight that the intensity, duration, and severity of undersaturation events exhibit high interannual variability along the central CCS shelfbreak. Variability in dissolved inorganic carbon (DIC) along the bottom of the 100-m isobath explains 70–90% of event severity variance over the range of latitudes where most severe conditions occur. An empirical orthogonal function (EOF) analysis further reveals that interannual event variability is explained by a combination coastal upwelling intensity and DIC content in upwelled source waters. Simulated regional DIC exhibits low frequency temporal variability resembling that of the Pacific Decadal Oscillation, and is explained by changes to water mass composition in the CCS. While regional DIC concentrations and upwelling intensity individually explain 9 and 43% of year-to-year variability in undersaturation event severity, their combined influence accounts for 66% of the variance. The mechanistic description of exposure to undersaturated conditions presented here provides important context for monitoring the progression of ocean acidification in the CCS and identifies conditions leading to increased vulnerability for ecologically and commercially important species.

## Introduction

The global ocean has absorbed approximately 25% of anthropogenic $CO_{2}$ emissions since the beginning of the industrial era^1^, resulting in ocean acidification. Rapid reductions in pH and carbonate ion $\left( {CO_{3}^{2 - } } \right)$ concentration in the upper ocean have emerged as pressing threats to marine life^2^. Of particular concern is decreasing calcium carbonate mineral $\left( {CaCO_{3} } \right)$ saturation states and its less stable polymorph aragonite. The saturation state of aragonite $\left( {{\Omega }_{{{\text{arag}}}} } \right)$ in seawater depends on the concentrations of calcium $\left( {\left[ {Ca^{2 + } } \right]} \right)$ and carbonate ions $\left( {\left[ {CO_{3}^{2 - } } \right]} \right)$, as well as the apparent solubility of aragonite $\left( {K^{\prime}_{{sp_{arag} }} } \right)$ at in situ temperature, salinity, and pressure, expressed as:

$${\Omega }_{{{\text{arag}}}} = \left[ {Ca^{2 + } } \right]\left[ {CO_{3}^{2 - } } \right] K^{\prime}_{{sp_{arag} }}$$

At ${\Omega }_{{{\text{arag}}}}$ less than 1, dissolution of aragonite becomes thermodynamically favorable, posing risk to organisms which use calcium carbonate minerals to build shells and skeletons.

The effects of ocean acidification are exacerbated in the California Current System (CCS) due to coastal upwelling^3,4^. In spring and summer, equatorward winds intensify along the west coast of North America. Upwelling favorable winds drive offshore transport of the surface Ekman layer, which is then replaced near the coast by waters originating from depths between 50 and 100 m^5–7^. These waters are rich in nutrients and both natural and anthropogenic CO_2_, making them simultaneously low in pH and ${\Omega }_{{{\text{arag}}}}$^7–9^. During upwelling events, enhanced nutrient content in upwelled water supports elevated primary production, which increases ${\Omega }_{{{\text{arag}}}}$ near the surface by drawing down CO_2_ levels, but also reduces ${\Omega }_{{{\text{arag}}}}$ at depth due to subsequent remineralization of production exported below the mixed layer. The addition of anthropogenic CO_2_ in a naturally low pH and low ${\Omega }_{{{\text{arag}}}}$ environment is expected to push conditions beyond biological thresholds, causing a variety of deleterious responses^2,10,11^. The depth at which ${\Omega }_{{{\text{arag}}}}$ becomes undersaturated, known as the ${\Omega }_{{{\text{arag}}}}$ saturation horizon, has already shoaled in nearshore waters of the CCS by 25–40 m since the preindustrial era, and corrosive waters have been observed on the continental shelf^7,12^.

Due to the intermittent nature of upwelling, whereby alongshore winds persisting for several days to weeks are followed by periods of relaxation, events of ${\Omega }_{{{\text{arag}}}}$ undersaturation are episodic in nature^4,13^. Such events have been captured in synoptic surveys as well as long-term time series, which documented exposure to corrosive conditions during periods of enhanced upwelling-favorable wind stress^7,14^. Alongshore variability in coastline topography also modulates atmospheric wind patterns and nearshore ocean circulation, thereby impacting the local magnitude of upwelling intensity, primary production, and pH on the shelf^15–19^. Furthermore, upwelling and primary production exhibit strong interannual variability due to changes in atmospheric and oceanic forcing associated with modes of climate variability, such as the El Niño Southern Oscillation (ENSO)^20,21^, Pacific Decadal Oscillation (PDO)^22,23^ and North Pacific Gyre Oscillation (NPGO)^24,25^. Due to their highly variable nature, the physical and biogeochemical drivers of low ${\Omega }_{{{\text{arag}}}}$ exposure in the CCS are typically difficult to fully interpret based on synoptic surveys and stationary time series. Coupled physical-biogeochemical ocean models provide a means to resolve variability at spatial and temporal scales observational platforms cannot simultaneously capture. Existing modeling studies have yielded important insights into the drivers of ocean acidification exposure in the CCS, and many have relied upon spatially-averaged metrics to focus on regional or basin scale processes shaping ocean acidification^3,26–30^. Exposure to low pH conditions (one of the important determinants of ${\Omega }_{{{\text{arag}}}}$ in the CCS) has been shown to exhibit strong alongshore heterogeneity, shaped by regional and local physical and biogeochemical processes, suggesting spatially-averaged metrics may obscure finer scale variability important to ocean acidification exposure^19^.

A framework to quantify low ${\Omega }_{{{\text{arag}}}}$ events by duration, intensity and severity was developed to characterize future exposure under a high emissions scenario using model output for the CCS^27^. The results highlighted that low ${\Omega }_{{{\text{arag}}}}$ events in the CCS will become more frequent and severe with increasing anthropogenic CO_2_ emissions. However, a comprehensive mechanistic understanding of the drivers of these events in a recent historical context is still lacking. The present study aims to address this gap using a ~ 3 km horizontal resolution ocean model spanning 1988–2010 to characterize the physical and biogeochemical drivers of ${\Omega }_{{{\text{arag}}}}$ undersaturation event intensity, duration, and severity at spatial and temporal scales relevant to marine ecosystem management. Since the rapid progression of OA along the U.S. west coast is projected to be spatially heterogeneous^19,28,30^, a comprehensive understanding of the mechanisms driving historical exposure to low ${\Omega }_{{{\text{arag}}}}$ provides valuable context for interpreting future intensity, duration and severity of exposure to undersaturation in the CCS due to anthropogenic climate change.

## Methods

### Model configuration

The physical circulation is resolved by the Regional Ocean Modeling System (ROMS)^31,32^. ROMS was configured with 42 terrain-following depth levels and a grid nesting approach consisting of an inner domain for the central CCS (32–44° N, 116.5–128.5° W) at 1/30° (~ 3 km) resolution forced by an outer domain for the broader CCS (30–48° N, 115–134° W) at 1/10° (~ 10 km) resolution with physical data assimilation. The physical circulation is forced at the surface by the 0.25° resolution Cross-Calibrated Multi-Platform (CCMP) winds^33^, and physical initial and boundary conditions for the outer domain are derived from the Simple Ocean Data Assimilation (SODA) reanalysis^34^. A complete description of the physical model configuration can be found in Fiechter et al.^18^.

Biogeochemical processes are simulated using NEMUCSC, an adaptation of the North Pacific Ecosystem Model for Understanding Regional Oceanography (NEMURO)^35^, with the addition of carbon and oxygen cycling based on formulations of Hauri et al.^27,28^, and Fennel et al.^36^, respectively. Boundary and initial conditions for the outer domain are derived from the World Ocean Atlas monthly climatology for nutrients and oxygen^37^, from the Global Ocean Data Analysis project for total alkalinity (TAlk)^38^, and from the empirical relationship of Alin et al.^39^ for dissolved organic carbon (DIC) based on monthly temperature and oxygen. Due to their climatological nature, the boundary conditions do not reflect global and basin-scale trends in biogeochemical properties. Atmospheric $pCO_{2}$ is prescribed based on the Mauna Loa time series, with a superimposed mean seasonal cycle and annual increase of 1.5 ppmv before 1995 and 2 ppmv thereafter. A complete description of the biogeochemical model configuration can be found in Cheresh and Fiechter^19^.

The analysis presented here is based on the higher-resolution inner domain at ~ 3 km resolution and focuses on the region between Pt Conception and Cape Blanco (35–43° N), which is known to exhibit substantial alongshore variability in physical and biogeochemical processes influencing pH (and presumably ${\Omega }_{{{\text{arag}}}}$ due to their strong covariability in the CCS)^20^. While the full simulation spans 1980–2010, the analysis is limited to 1988–2010 to eliminate spin-up effects.

### Model evaluation

The model is evaluated with observations from the 2007 West Coast Ocean Acidification (WCOA) Cruise between 35 and 42° N by comparing simulated daily mean values for May–June 2007 to synoptic measurements from 0 to 300 m depth at the 4 stations closest to shore along each transect. The ability of the model to reproduce temporal and alongshore properties of undersaturation events is also evaluated using daily-averaged model output compared to daily-averaged observations from nearshore moorings deployed at approximately 12-m depth within kelp forests at ~ 35.2, 36.0, 38.9, and 39.3° N^40^. Because the observations (2017–2019) and simulation (1988–2010) do not overlap in time, the model is evaluated for its ability to reproduce the mean, range, and monthly climatological properties of measured pH, temperature, and dissolved oxygen (DO). An event analysis (as described below) is applied to simulated and observed variables to further assess the capacity of the model to capture low pH events (pH < 7.7, which approximates a mean ${\Omega }_{{{\text{arag}}}}$ of 1 over a wide range of TAlk values regularly observed in the CCS (i.e., 2100–2300 μmol/kg).

### Event analysis

The event analysis focuses on determining the properties of undersaturated events with respect to aragonite, where “events” are defined as periods of time when ${\Omega }_{{{\text{arag}}}}$ values are lower than a specified threshold. Following Hauri et al.^27^, events are characterized by their intensity, duration, and severity. Duration (D) represents the number of consecutive days ${\Omega }_{{{\text{arag}}}}$ values are below the threshold during an event. Intensity (I) represents the difference between the threshold (T) and the mean saturation state (${\Omega }_{{{\text{mean}}}}$) during an event (*I* = *T−*${\Omega }_{\mathrm{mean}}$). Severity is quantified as the product of duration and intensity (*S* = *D × I*). Although biological thresholds are presumably species- or even population-specific, a heuristic threshold of ${\Omega }_{{{\text{arag}}}}$ = 1 is chosen here, as exposure to saturation states below 1 thermodynamically favors dissolution of calcium carbonate.

Intensity, duration, and severity are calculated using daily averaged model fields extracted along the 100-m isobath as a location characteristic of alongshore, seasonal, and interannual event variability affecting coastal waters in the central CCS. The bottom of the 100-m isobath is also indicative of upwelled source water properties^21^ and, therefore, of the conditions that eventually modulate coastal exposure to undersaturated events. Physical and biogeochemical water mass properties associated with events are determined by averaging annually over days during which conditions along the bottom of the 100-m isobath were undersaturated. These properties are then individually regressed against event severity to identify variables most closely associated with interannual variability in event properties at each latitude. While averaged meridional wind stress within 50 km from the coast is included in the regression as a proxy for coastal upwelling intensity^18^, the influence of regional upwelling dynamics and water mass properties is assessed more extensively by examining the depth and physical and biogeochemical properties associated with the 26.0 kg/m^3^ isopycnal (${\sigma }_{26}$), a density surface historically considered as representative of upwelled source waters in the central CCS^41^. An EOF analysis is used to isolate the dominant modes of regional variability for meridional wind stress, as well as the depth and DIC content of ${\sigma }_{26}$ within 200km from the coast (a representative offshore distance over which regional variability relates to changes in upwelled source waters). Finally, a multiple linear regression model is used to relate the variables identified as important in the analysis to interannual variability of event severity. All analyses are conducted and figures created using Ferret v7.1 (http://ferret.pmel.noaa.gov/Ferret/), except for the multiple linear regression and associated statistical tests done using R (stats, lmtest and zoo packages)^42–45^.

## Results

### Model evaluation

**Figure 1:**
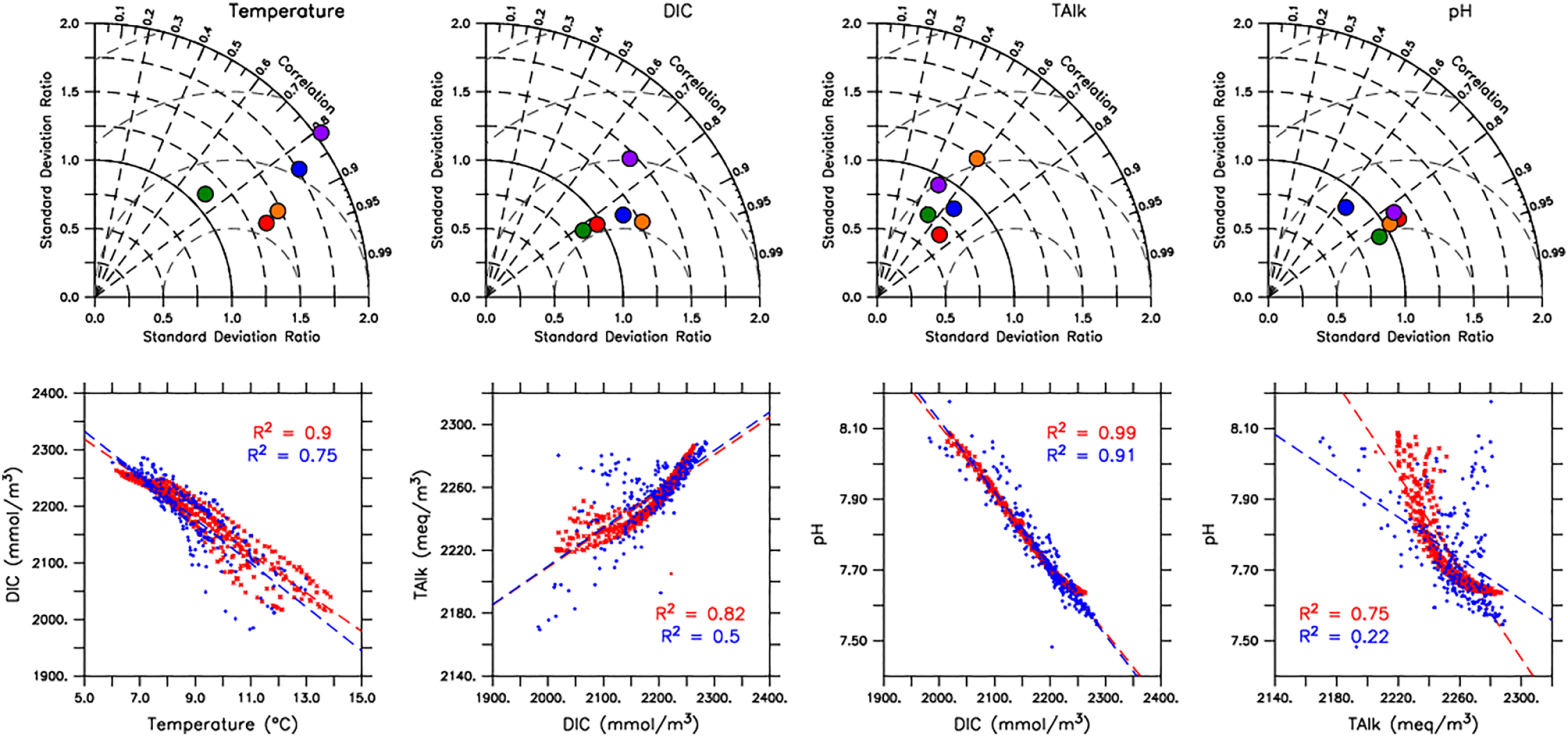
Simulated and observed conditions during the 2007 WCOA Cruise. Top panels: Taylor diagrams for (from left to right) temperature, DIC, TAlk, and pH along transect lines at 35° N (purple), 37° N (orange), 38° N (blue), 40° N (green), and 42° N (red). Radial distance corresponds to the ratio of simulated to observed standard deviations and azimuthal angle corresponds to model-observation correlation. Dashed concentric circles centered at (1, 1) denote root mean square difference normalized by observed standard deviation (0.5 increments). Bottom panels: simulated (red) and observed (blue) relationships for (from left to right) temperature vs. DIC, DIC vs. TAlk, DIC vs. pH, and TAlk vs. pH. Dashed lines indicate least-square linear fit for model and observations and corresponding ${\mathrm{R}}^{2}$ values are listed in each panel. Both Taylor diagrams and regressions are based on the 4 stations closest to the coast along each transect line and restricted to values in the 0–300 m depth range. Observations are synoptic values and simulated values represent means over the cruise period for the Taylor diagram and daily means for the regressions.

The ability of the simulation to reproduce observed variability across the shelf at several latitudes spanning the model domain is evaluated in terms of the agreement between observed and simulated properties via Taylor diagrams (Fig. 1, upper panels), and between observed and simulated relationships for two properties via linear regressions (Fig. 1, lower panels). Based on the Taylor diagrams, simulated DIC and pH exhibit the closest agreement with in situ measurements, with standard deviation ratios (modeled to observed) of 0.75–1.25 and correlation coefficients above 0.8 at most locations, resulting in root mean square differences (RMSD) of 0.5–1 observed standard deviation (i.e., RMSD are less than 1 observed standard deviation and often close to 0.5 standard deviation). Simulated temperatures correlate well with observations (r > 0.7), but are more variable (standard deviation ratios of 1–2), yielding RMSD between 0.5 and 1.25 observed standard deviations. Simulated and observed TAlk values are more weakly correlated (r = 0.3–0.75), but have comparable variability (standard deviation ratios between 0.5 and 1.25), leading to RMSD in the range of 0.5–1 observed standard deviation. A summary of the statistics for each variable and latitude, along with model bias, is provided in Supp. Table 1. A least squares regression between temperature and DIC yields R^2^ values of 0.75 for the observations, and 0.85 for the model. R^2^ values for a regression between DIC and pH are higher, with 0.91 for observed and 0.99 for simulated values. Regressions of TAlk with DIC and pH reveal weaker relationships, with respective R^2^ values of 0.5, 0.22 for the observations, and 0.85 and 0.75 for the model. These comparisons indicate that in both model and observations, DIC explains a substantially greater fraction of pH (and presumably saturation state) variability than TAlk over the coastal region and depths considered here. Overall, the model evaluation demonstrates reasonable agreement between simulated and observed variables, especially for DIC and pH, across the shelf at latitudes (35–42° N) spanning the central CCS. The assessment is consistent with that of Cheresh and Fiechter^19^ based on the same model output and in situ observations, which identified that biogeochemical discrepancies were generally associated with physical conditions not exactly matching the synoptic timing of upwelling along transects.

**Figure 2:**
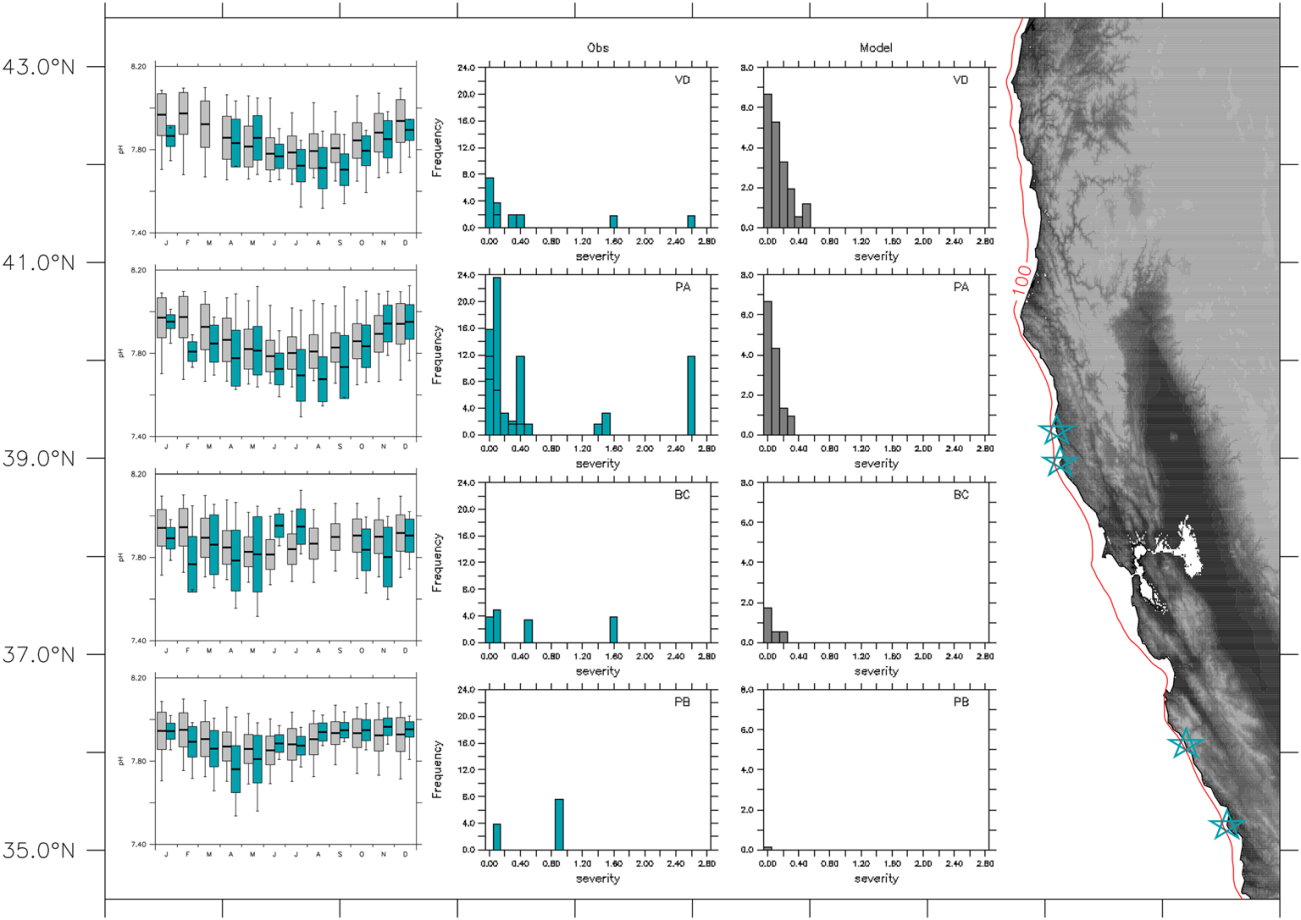
Simulated (1988–2010) and observed (2017–2019) pH at nearshore moorings indicated by blue stars on map at right (Van Damme (39.27° N, − 123.80° W), Point Arena (38.95° N, − 123.74° W), Big Creek (36.0° N, − 121.60° W) and Point Buchon (35.24° N, − 120.9° W); red line denotes 100-m isobath). (Left) Monthly climatological mean, standard deviation and minimum/maximum values represented by box and whisker plots for observed (blue) and simulated (gray) pH values. (Middle and Right) Frequency histograms of observed and simulated severity of low pH events (pH < 7.7) per year at each location. Map created using Ferret v7.1 (http://ferret.pmel.noaa.gov/Ferret/).

For nearshore moorings, in situ pH values generally fall within the range of simulated climatological values, and simulated mean climatological pH values track the observed seasonal cycle at all locations (Fig. 2). The observed climatological monthly mean falls within simulated minimum and maximum climatological values. In most cases, the observed monthly mean is contained within one simulated climatological standard deviation from the model monthly climatological mean. The model’s ability to reproduce monthly pH variability (in a climatological sense) also extends to temperature and dissolved oxygen (Supp. Fig. 1). Frequency histograms of low pH (< 7.7) event severity at each site indicate that simulated and observed range and distribution of severity values are consistent, with the highest proportion of events having low severity (Fig. 2). Furthermore, the alongshore pattern in frequency distributions for event severity is similar between model and observations, with more frequent and higher severity events occurring at northern sites (Van Damme and Point Arena) relative to southern sites (Big Creek and Point Buchon). Because severity is calculated using intensity and duration, the model-data agreement extends to these properties as well (Supp. Fig. 2).

### Spatiotemporal variability of undersaturation events

**Figure 3:**
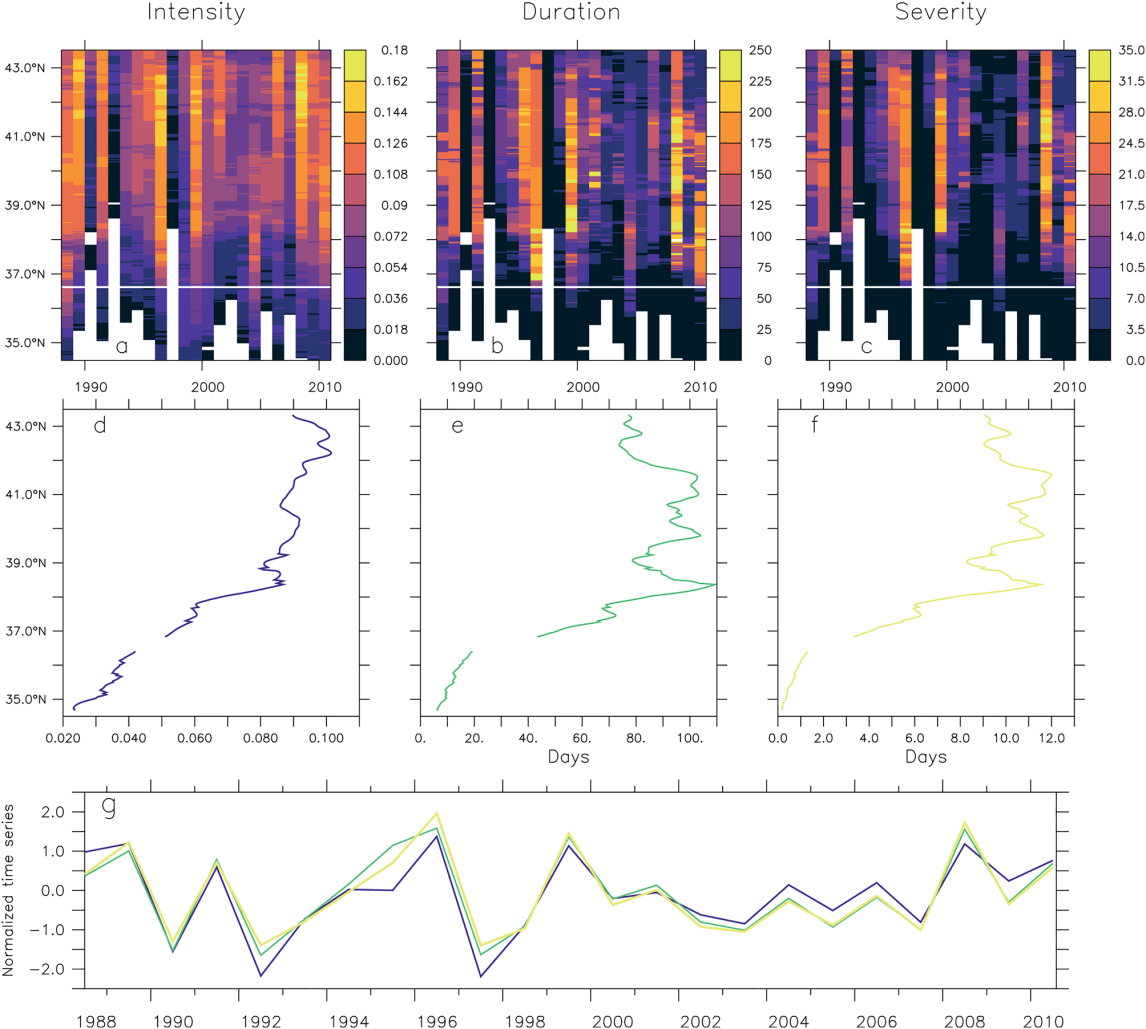
Simulated aragonite undersaturation (${\Omega }_{{{\text{arag}}}}$ < 1) event properties during the upwelling season (May–September) along the bottom of the 100-m isobath. (Top) Annual mean intensity (**a**), duration (**b**) and severity (**c**). (Middle) 1988–2010 mean event intensity (**d**), duration (**e**) and severity (**f**) as a function of latitude. (Bottom) Standardized annual mean event intensity (blue), duration (green) and severity (yellow) averaged over latitude (34.5–43.5° N).

Exposure to undersaturated conditions along the 100-m isobath varies strongly with depth, latitude, and time (Fig. 3 and Supp. Fig. 3). Near the surface, there are locations where no events occur throughout the entire simulation; where events occur, they typically have low intensity, short duration, and low severity. At depths below 60 m, events occur at every latitude and are most intense, longest, and most severe near the bottom.

Along the bottom of the 100-m isobath, exposure to undersaturated conditions in the central CCS is most significant during the period of strongest coastal upwelling favorable conditions (May–September, hereafter referred to as “upwelling season”) (Supp. Fig. 3). Event properties along the bottom of the 100-m isobath exhibit strong interannual variability, with years of low intensity, duration and severity (e.g., 1992 and 1997), and years experiencing prolonged undersaturation (e.g., 1999 and 2008) (Fig. 3a–c). Based annual upwelling season averages, intensity ranges from 0.0 to 0.2 (corresponding to average ${\Omega }_{{{\text{arag}}}}$ of 1.0–0.8), and average duration and severity ranges from 1 to 258 days and 0–34 days, respectively. While simulated events occur at all latitudes, they are more intense, longer lasting, and therefore more severe, north of 37°N. The alongshore and temporal properties of these events also indicate a strong covariability of intensity, duration, and severity (Fig. 3d–g).

**Figure 4:**
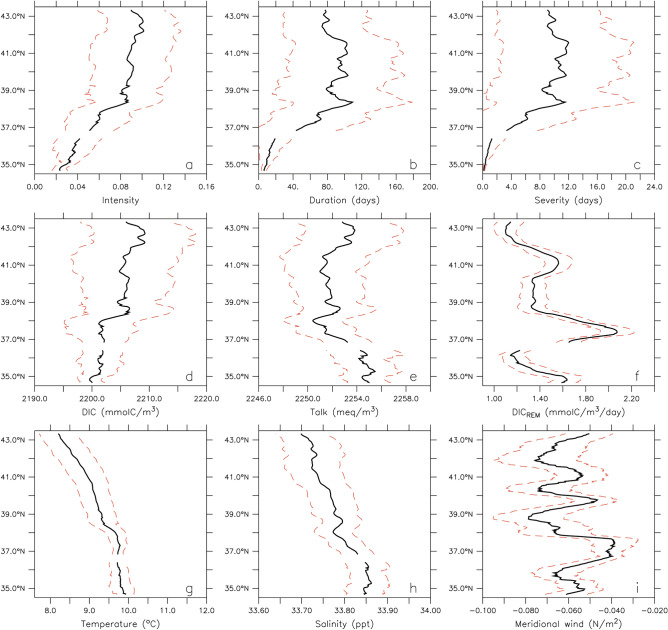
Upwelling-season mean (black lines) and standard deviation (red dashed lines) of event properties and associated water mass characteristics during events (i.e. when Ω_arag_ < 1). (Top) Event intensity (**a**), duration (**b**), and severity (**c**). (Middle) DIC (**d**), TAlk (**e**), and rate of DIC production from remineralization (${\mathrm{DIC}}_{\mathrm{REM}}$) (**f**). (Bottom) Temperature (**g**), salinity (**h**), and meridional wind stress averaged 0–50 km offshore (**i**).

**Figure 5:**
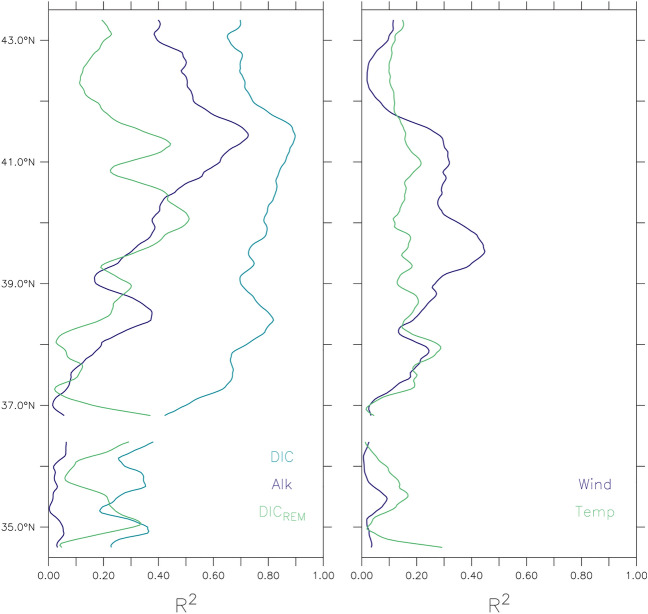
Relationships between event severity and water mass properties during undersaturated conditions (${\Omega }_{{{\text{arag}}}}$ < 1) along the bottom of the 100-m isobath based on upwelling season means. (Left) R^2^ values between severity and DIC (blue line), severity and total TAlk (green line), and severity and DIC_REM_ (purple line). (Right) R^2^ values between severity and meridional wind stress (purple line) and severity and temperature (green line). All values are from the bottom of the 100-m isobath, except meridional wind stress, which represents a 0–50 km offshore average.

Temporal means and standard deviations demonstrate that interannual variability greatly exceeds (by as much as a factor of 2–3) alongshore variations over the region where undersaturated conditions occur most frequently (i.e., 38–43° N). Average water mass properties during undersaturated conditions along the bottom of the 100-m isobath indicate alongshore similarities between event properties and DIC and TAlk (Fig. 4). In contrast, alongshore patterns of temperature, salinity, and DIC contribution from remineralization (${\mathrm{DIC}}_{\mathrm{REM}}$) bear less resemblance with event properties. Pairwise linear regressions between event severity and water mass properties during events indicate that DIC explains the largest fraction of the interannual variability in event severity (R^2^ of 0.7–0.9 in regions of high event severity north of 37° N) (Fig. 5, left panel). In contrast, TAlk and DIC_REM_ independently explain less than 50% of interannual variance in event severity, except for a narrow range of latitudes between 40 and 41° N where R^2^ values for TAlk increase to ~ 0.7 (but still lower than the R^2^ values for DIC of ~ 0.9 over the same range of latitudes). For physical variables, temperature is generally not a good predictor of event severity (R^2^ < 0.3) and the explanatory power of meridional (upwelling favorable) wind stress is limited to latitudes ranging between 38 and 41° N and explains 30–50% of the interannual variance in event severity (Fig. 5, right panel).

The emergence of DIC as the dominant driver of year-to-year variations in undersaturated conditions along the bottom of the 100-m isobath at all latitudes based on the regressions is consistent with the strong simulated and observed relationship between DIC and pH (and presumably ${\Omega }_{{{\text{arag}}}}$) in coastal waters of the central CCS (see Fig. 1). While TAlk, DIC_REM_, and meridional wind stress can locally explain a non-trivial fraction of the event severity variance, their importance consistently falls below that of DIC. The dominance of DIC as the driver of interannual variability in ${\Omega }_{{{\text{arag}}}}$ is further confirmed with a sensitivity study following Turi et al. 2018 (Supp. Table 2). DIC explains on average 75% of the interannual variability (approximately 80% at 35° N decreasing roughly linearly to 70% at 43° N), while TAlk explains roughly 20% of variability in ${\Omega }_{{{\text{arag}}}}$ (Supp. Fig. 4). Temperature has a small (~ 5%) impact on ${\Omega }_{{{\text{arag}}}}$, and salinity’s contribution is negligible.

DIC and TAlk budgets were also constructed to identify the physical (advection and mixing) and biogeochemical (remineralization, calcium carbonate dissolution, and nitrification) processes controlling changes in these properties along the bottom of the 100-m isobath. Advection emerges as the dominant mechanism influencing interannual variability in DIC and TAlk during the upwelling season, with average contributions at most latitudes greater than any other process by at least two orders of magnitude (Supp. Fig. 5).

### Drivers of undersaturation events

Possible mechanisms through which DIC concentrations along the bottom of the 100-m isobath can vary interannually are: (1) changes in upwelling intensity, whereby weaker or stronger upwelling would lead to lower or higher DIC concentrations, and (2) changes in DIC concentrations near the source depth of upwelled water independent of upwelling intensity.

**Figure 6:**
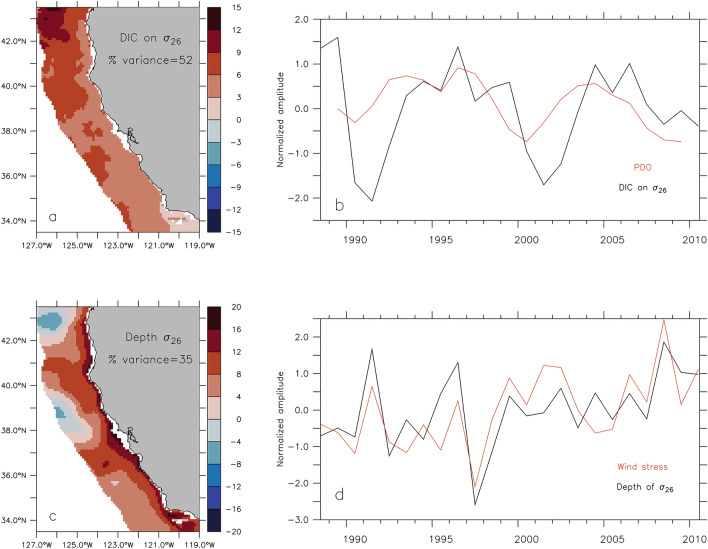
Leading EOF mode of interannual variability for the DIC content (top) and depth of ${\sigma }_{26}$ (bottom) based on upwelling season averages and restricted to 0-200km offshore. (**a**) Spatial patterns of EOF mode 1 for ${\sigma }_{26}$ DIC content (mmolC/m^3^). (**b**) Temporal amplitude of EOF mode 1 for ${\sigma }_{26}$ DIC content (black line), and 3-year running mean of Pacific Decadal Oscillation (PDO) index (red line) (https://www.ncdc.noaa.gov/teleconnections/pdo/). (**c**) Spatial pattern of EOF mode 1 for ${\sigma }_{26}$ depth (m); positive values indicate shallower depths. (**d**) Temporal amplitude of EOF mode 1 for ${\sigma }_{26}$ depth (black line) and temporal amplitude of EOF mode 1 for meridional wind stress averaged within 50 km of the coast (red line). Maps created using Ferret v7.1 (http://ferret.pmel.noaa.gov/Ferret/).

To explore the relative importance of changes in upwelling intensity vs. changes in upwelled source water DIC content (associated with anomalous transport of water masses), regional variations of the two following properties are considered: (1) depth of ${\sigma }_{26}$ as a proxy for changes in upwelling intensity, and (2) DIC concentrations on ${\sigma }_{26}$ as a proxy for changes in upwelled source water properties. The depth of ${\sigma }_{26}$ is commonly used in the central CCS to track the source depth of upwelled waters and upwelling strength, and is approximately 50–60 m during the upwelling season along the 100-m isobath (Supp. Fig. 6). The first EOF mode for DIC concentrations on ${\sigma }_{26}$ explains 52% of the variance and is characterized by a broad, regional pattern with higher variability in the northern part of the domain (i.e., where events are most severe) (Fig. 6). The temporal amplitude of this mode exhibits primarily low-frequency variability, with positive and negative anomalies lasting ~ 3–7 years and resembling the Pacific Decadal Oscillation. Positive DIC anomalies correspond with positive phases of the PDO, and vice versa, although with an apparent lag of ~ 1 year. The first EOF mode for the depth of ${\sigma }_{26}$ explains 35% of the variance and exhibits a predominantly coastal signal. The temporal variability of this mode is dominated by interannual variability and aligns with the temporal amplitude of the first EOF mode for meridional wind-stress averaged within 50 km of the coast.

**Figure 7:**
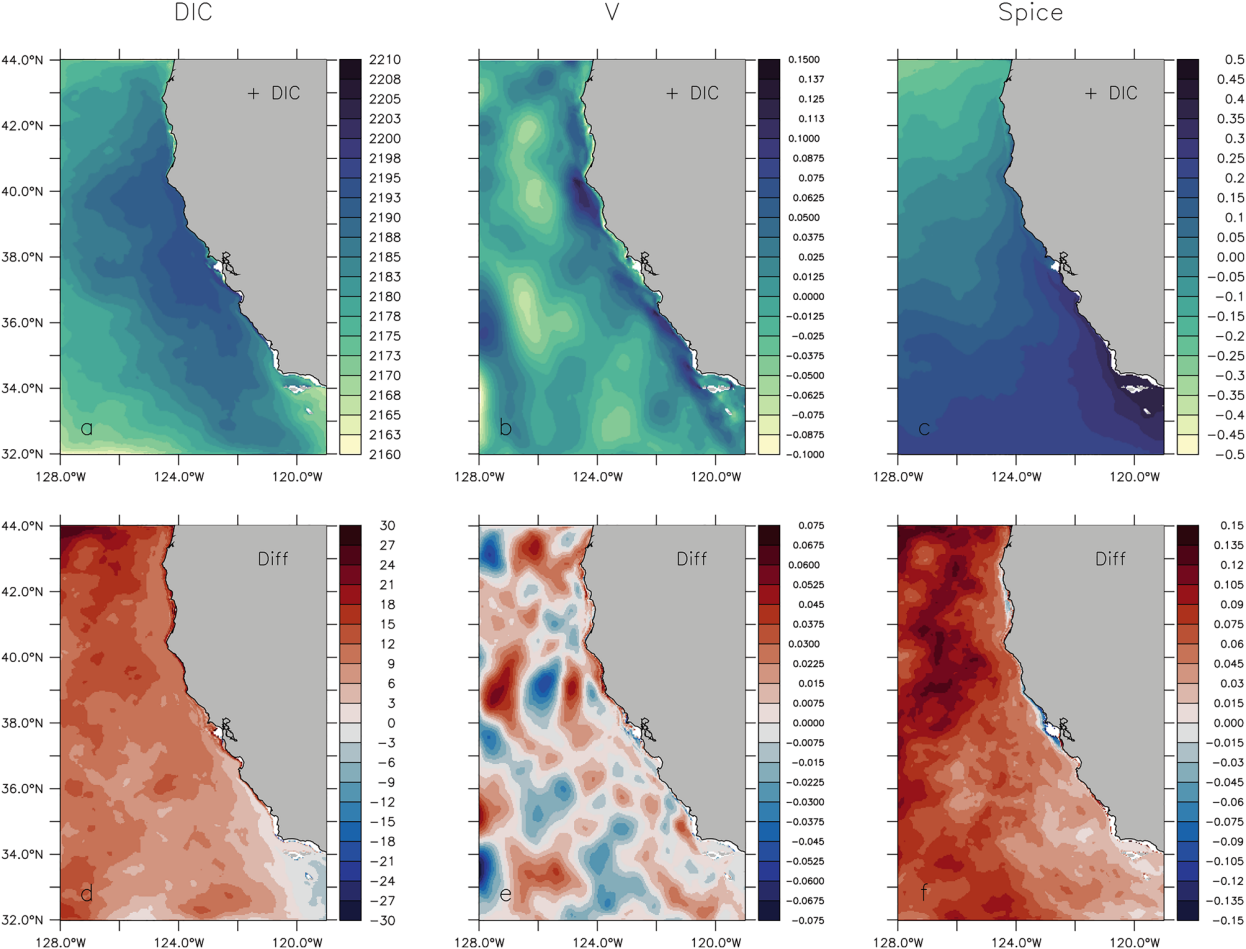
Environmental conditions during periods of increased DIC content on ${\sigma }_{26}$. (Top) Upwelling-season mean DIC concentrations (mmolC/m^3^) (**a**), meridional velocity (m/s) (**b**), and spice (kg/m^3^) (**c**) on ${\sigma }_{26}$ during positive phases of the first EOF mode for DIC. (Bottom) Upwelling-season mean DIC (mmolC/m^3^) (**d**), meridional velocity (m/s) (**e**), and spice (kg/m^3^) (**f**) differences between positive and negative phases of the first EOF mode for DIC. Maps created using Ferret v7.1 (http://ferret.pmel.noaa.gov/Ferret/).

While the first EOF mode for the depth of ${\sigma }_{26}$ is clearly related to meridional wind stress and interannual variations in upwelling intensity (i.e., shallower isopycnal depths occur during years of stronger upwelling), the regional patterns associated with the first EOF mode for DIC on ${\sigma }_{26}$ warrant further examination. The low frequency variability of the mode, and apparent relationship to the PDO, suggest a connection to changes in the intensity of the subtropical gyre circulation and water mass advection into the central CCS. Upwelling-season mean meridional velocities and spice calculated at the depth of ${\sigma }_{26}$ and separately averaged for years of positive and negative amplitudes of the first EOF mode for DIC (see Fig. 6b) verify this hypothesis. During positive phases of the first EOF mode, DIC at the depth of ${\sigma }_{26}$ is, as expected, higher over the entire region (Fig. 7a). The difference in DIC concentrations between positive and negative phases ranges from approximately − 5 to 30 mmolC/${\mathrm{m}}^{3}$ (Fig. 7d). The largest positive differences occur north of 37° N, which is consistent with the spatial patterns of mode 1 indicating the highest variability in the northern part of the domain (see Fig. 6a). Similar differences (positive vs. negative DIC phases) for meridional velocities and spice (Fig. 7b, c, e, f) clearly indicate that the regional increase in DIC concentrations is associated with an equivalent increase in poleward velocities and spice on ${\sigma }_{26}$, pointing to a strengthening of northward transport of Pacific Equatorial Waters (which have higher spice and DIC content on ${\sigma }_{26}$ than Pacific Subarctic Upper Waters) in the central CCS. An increased contribution of Pacific Equatorial Waters to the region is also consistent with a weakening of the subtropical gyre circulation and, therefore, a positive phase of the PDO.

The combined effect of low frequency changes in DIC content near the depth of upwelled source waters and higher frequency variability associated with interannual changes in coastal upwelling intensity on undersaturated event properties along the bottom of the 100-m isobath is further quantified via a multiple linear regression (MLR). Using upwelling season alongshore mean values for the depth of ${\sigma }_{26}$ (as a proxy for upwelling intensity) and DIC concentrations on ${\sigma }_{26}$ (as a proxy for upwelled source water content) as explanatory variables and log severity as a response variable, a MLR given by log(severity) = − 195.06754 + 0.12333 (${\sigma }_{26}$) + 0.09645 ([DIC]) yields a multiple R^2^ value of 0.66, and a p-value of 1.967e−05 (Supp. Fig. 5). Regressions between event severity and the two explanatory variables indicate that, individually, the depth of ${\sigma }_{26}$ explains 43% of interannual variability in severity, and DIC concentrations on ${\sigma }_{26}$ explains 9% of interannual variability in severity (Supp. Table 1).

## Discussion

Previous work has identified a region-wide susceptibility to ocean acidification in the CCS, and the present study describes recent spatiotemporal patterns and drivers of exposure to undersaturated conditions. The results demonstrate that event intensity, duration, and severity in the central CCS exhibit substantial interannual variability governed by changes in the strength of coastal upwelling and DIC concentrations in upwelled source waters.

Comparison of simulated pH, dissolved oxygen, and temperature against observed data from nearshore moorings indicates the model reproduces, in a climatological sense, natural variability experienced in coastal waters on monthly time scales. Episodic excursions of observed pH below the minimum simulated values are attributed to a model bias towards higher pH levels near the source depth of upwelled water identified in previous evaluations^19^. The tendency of the model to simulate slightly higher than observed pH values suggests that the present assessment of exposure to undersaturated conditions is likely conservative and provides lower bounds for event properties. While noticeable differences exist in the magnitude of pH values, the alongshore pattern of event occurrence and overall magnitude of event properties are generally consistent between the model and observations. The focus of the present study is to identify dominant drivers of interannual event variability rather than provide exact estimates of event properties in terms of their magnitude. As such, the interpretation relies on regional spatial and temporal patterns to describe event properties along the 100-m isobath, while in reality there are additional physical and biogeochemical mechanisms, such as tidal mixing and benthic processes, that will further influence event properties at the local scale of nearshore environments. The lack of a global anthropogenic signal in the model boundary conditions and the relatively short simulation period also preclude any conclusions about trends in corrosive events from the present analyses.

A distinct separation between the northern and southern regions of the central CCS is apparent in the alongshore pattern of event properties, such that intensity, duration, and severity values are lower south of 37° N, and higher north of 37° N. The region of higher event severity north of 37° N corresponds to latitudes experiencing overall higher mean DIC concentrations and lower mean TAlk relative to the region south of 37° N. While DIC is the variable most closely associated with interannual variability in event severity over the range of latitudes considered here, other variables, such as TAlk, organic matter remineralization, and meridional wind stress, may locally influence the alongshore pattern and temporal variability of event properties. For example, the fraction of interannual variance in event severity explained independently by TAlk and remineralization substantially increases in the region of higher primary production south of Cape Blanco (41–42° N; as identified in Cheresh and Fiechter^19^ and evidenced by higher POC remineralization rates in Fig. 4). The importance of DIC over TAlk in shaping the interannual response of ${\Omega }_{{{\text{arag}}}}$ was confirmed with a sensitivity analysis, and is consistent with Turi et al.^46^, who also found DIC to be the dominant variable driving changes in pH within 100 km from the coast in the central CCS. The results presented here do not contradict previous work identifying the role of remineralization in reducing ${\Omega }_{{{\text{arag}}}}$ at depth in the CCS^9^, but rather establish that it has limited explanatory power for year-to-year variability in event intensity. Based on the interannual variability of simulated remineralization rates during events at the bottom of the 100-m isobath (standard deviation of ~ 0.2 mmolC/${\mathrm{m}}^{3}$/day; Fig. 4), it would take the full average duration of an event (~ 80 days; Fig. 4) to create a DIC change comparable to the interannual DIC variability during events (standard deviation of ~ 10 mmolC/${\mathrm{m}}^{3}$; Fig. 4). Furthermore, when DIC content in upwelled source waters is regionally elevated, contributions from remineralization within 200 km of the coast are on average lower (Supp Fig. 4), suggesting a reduction in the supply of DIC from export production and a mitigating effect on undersaturation events. DIC and TAlk budgets were also constructed to dissociate physical and biogeochemical contributions, and revealed that advection is the dominant process (by at least two orders of magnitude) impacting the interannual variability of DIC and TAlk during the upwelling season along the bottom of the 100-m isobath. While this finding confirms that interannual variations in coastal upwelling intensity and DIC content in upwelled source waters are the primary processes controlling the onset and intensity of undersaturation events during the upwelling season, remineralization may prolong the duration of events at alongshore locations where enhanced primary production and vertical export occur. Additional mechanisms may also shape the alongshore properties of events at finer spatial scales, such as variations in upwelling intensity and duration as well as regional circulation patterns^19^.

Based on the leading EOF mode for DIC concentrations on ${\sigma }_{26}$ within 200 km of the coast, more than half of DIC variance is related to a low-frequency, regional signal that contributes differences up to 30–40 mmolC/${\mathrm{m}}^{3}$ between periods of increased and decrease DIC (Figs. 6 and 7) in the northern domain. The temporal amplitude of this EOF mode resembles the Pacific Decadal Oscillation index. When the EOF amplitude and PDO index are positive, higher DIC concentrations occur on ${\sigma }_{26}$ in combination with spicier waters and increased northward velocities. The connection to the PDO and co-occurrence of higher DIC concentrations, higher spice values, and increased poleward transport at depth point toward a change in water mass composition of the central CCS. This interpretation is consistent with a previous water mass analysis based on observations collected near 34.5° N, which concluded that 1996 (a year with higher simulated DIC content) had a lower fraction (~ 40%) of PSUW at 100-m depth, while 2008 (a year with lower simulated DIC content) had a higher fraction (~ 70%) of PSUW at the same depth^45^. While a temporal lag is expected between change in the intensity of the subtropical gyre circulation and changes in water mass composition in the coastal regions of the central CCS, the 1-year lag between the PDO and simulated DIC concentrations on ${\sigma }_{26}$ (Fig. 6) may be an artifact of averaging annually, such that any lag would be artificially amplified to one year.

**Figure 8:**
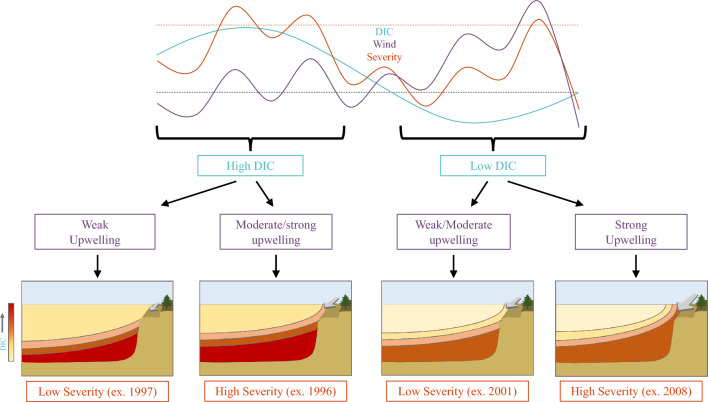
Conceptual flow diagram of undersaturation (${\Omega }_{\mathrm{arag}}$< 1) event severity and its drivers in the central California Current System. (Top) Theoretical time series of dissolved inorganic carbon (DIC) concentration (blue), upwelling-favorable wind stress (purple) and event severity (orange), with the dashed gray and dashed orange lines representing a theoretical mean and one standard deviation, respectively, and the shaded orange regions representing “extreme” events. (Bottom) Conceptual cross-sections of the upwelling-dominated coastal region, with gray lines indicating theoretical density surfaces, color shading indicating concentration of DIC, and gray alongshore arrow size representing the relative strength of upwelling-favorable winds.

The regional patterns and variability of DIC content in upwelled source waters cannot alone explain event severity, however. For example, in 1997, anomalously low event severity occurred alongside above average regional DIC concentrations. In contrast, simulated events in 2008 were anomalously severe, despite average regional DIC concentrations. Therefore, it is not only the DIC content in upwelled source waters, but also the strength of upwelling that dictates event severity. The first EOF mode for the depth of ${\sigma }_{26}$ exhibits a coastal signature with high-frequency temporal variability associated with the leading mode of variability for meridional (upwelling-favorable) wind stress, thereby suggesting that interannual variability in the depth of ${\sigma }_{26}$ within 200 km of the coast is primarily governed by changes in coastal upwelling intensity. When regional DIC is high and average to above average winds occur (e.g., 1996 and 1999), high DIC waters are upwelled and severe events happen. However, elevated regional DIC superimposed to below average upwelling intensity is not by itself sufficient to produce undersaturated conditions along the shelfbreak. This finding suggests that, based on the 1988–2010 period, average or above average upwelling-favorable wind stress is a necessary condition for high severity events to occur. In contrast, anomalously strong upwelling-favorable wind stress alone can lead to severe events despite below average DIC concentrations in upwelled source waters (i.e., during a negative PDO phase). These results highlight the various pathways by which low to high severity events can occur in the central CCS (Fig. 8).

It is worth noting that during positive phases of the PDO, when regional DIC concentrations are elevated, upwelling-favorable wind stress tends to be weaker (Fig. 6), which is consistent with reductions in upwelling strength during positive phases of the PDO, and vice versa^24^. Over the 23-year simulation, there are few instances when high regional DIC co-occurred with strong upwelling conditions, suggesting a potential compensatory effect of reduced upwelling during years of anomalously high regional DIC, theoretically mitigating the severity of undersaturation events in the central CCS.

Turi et al.^46^ established a relationship between basin-scale climate variability and nearshore pH variability in the CCS. Specifically, ENSO variations were linked to changes in water mass composition and primary productivity, which regionally modulated exposure to low pH. Turi et al.^46^ also demonstrated that La Niña events correspond to anomalously low pH and DIC below ~ 100 m. While the present results corroborate that ENSO variability plays a role in driving interannual variability in event severity (e.g., 1989, 1996, 1999, and 2008 all have above average event severity and correspond with moderate to strong La Niña conditions), it cannot by itself explain the simulated interannual variability in event severity. For example, moderate to strong La Niña events occurred in 2000, 2006 and 2009, but resulted in below average simulated event severity, suggesting that a moderate to strong La Niña event (and associated changes in water mass composition and coastal upwelling intensity) may not solely dictate undersaturation events at 100-m depth. It is more likely that interannual modes of variability modulating physical and biogeochemical properties in the central CCS, such as ENSO, superimpose on lower frequency modes of basin-scale variability, such as the PDO, and potentially exacerbate or mediate exposure to undersaturated conditions.

The present study builds on the work of Hauri et al.^27^, in which changes to simulated event properties from the preindustrial era through 2050 were quantified under a high-emissions scenario. Event properties calculated here are higher than those reported in Hauri et al.^27^ for the 5-year time period approximating 2010. The discrepancy could be attributed to wind forcing differences between the two simulations. Hauri et al.^27^ used climatological wind forcing, which likely underestimates synoptic variability and, therefore, the intensity of coastal upwelling events. Aditionally, Hauri et al.^27^ reported a model bias towards higher ${\Omega }_{{{\text{arag}}}}$ and noted that this could lead to an underestimation of event duration. Furthermore, the present analysis focuses on events along the bottom of the 100-m isobath, whereas Hauri et al.^27^ included shallower shelf regions where events may be shorter and less intense, reducing spatially-averaged event property statistics.

The current findings have important implications for marine species and ecosystems in upwelling regions. In the CCS, exposure to undersaturated conditions has been linked to shell or carapace dissolution of ecologically important species^10,11^. Most observational studies examining the ecological effects of ocean acidification have necessarily related snapshots of organisms’ conditions to concurrent conditions. However, the duration, intensity and severity of exposure is likely important in governing species’ responses. The effects of exposure to low ${\Omega }_{\mathrm{arag}}$ and pH conditions are likely to differ with intensity (e.g., increasingly deleterious effects with increasing intensities^47^). Thus, the results detailed here add to a growing body of evidence that the impacts of ocean acidification along the U.S. west coast will not be experienced uniformly with latitude and vary significantly from year to year. The mechanistic exploration of the drivers of events in high-resolution model simulations provides a valuable framework for identifying geographic hotspots or oceanographic conditions (e.g., PDO) associated with increased vulnerability to ocean acidification at local and regional scales.

### Supplementary Information

Supplementary Information.

## Data Availability

The model output used to complete the analysis has been deposited on Dryad (10.7291/D1M97Z). The observational data will be made available on the data platform for CenCOOS (https://data.caloos.org/). Contact the corresponding author to request additional information about or access to the datasets used in this study.
