# Supplementary material for: Upwelling intensity and source water properties drive high interannual variability of corrosive events in the California Current
Source: Sci Rep. 2023 Aug 10;13:13013. doi: 10.1038/s41598-023-39691-5 (PMC10415283; doi:10.1038/s41598-023-39691-5)
Supplement: Supplementary file 1 — Supplementary Information. [file 41598_2023_39691_MOESM1_ESM.docx]

*S**cientific Reports*

Supplementary Information for

**Upwelling intensity and source water properties drive high interannual variability of corrosive events in the California Current**

${Julia Cheresh}^{1,*}$, ${Kristy Kroeker}^{2}$, ${Jerome Fiechter}^{1}$

1 University of California, Santa Cruz, Department of Ocean Sciences, Santa Cruz, 95060, USA

2 University of California, Santa Cruz, Department of Ecology & Evolutionary Biology, Santa Cruz, 95060, USA

Contents of this file

Supplementary Table 1 – Summary of model-data statistics

Supplementary Table 2 – Carbonate system sensitivities

Supplementary Table 3 – Multiple linear regression model selection

Supplementary Figure 1 – Model-data comparison for mooring DO and temperature

Supplementary Figure 2 – Model-data comparison for mooring event duration and intensity

Supplementary Figure 3 – Latitude-depth and monthly climatology of event properties

Supplementary Figure 4 – Latitude-time DIC, alkalinity, temp and salinity contributions to $\Omega_{\mathrm{arag}}$

Supplementary Figure 5 – DIC and alkalinity budgets

Supplementary Figure 6 – Depth of $\sigma_{26}$

Supplementary Figure 7 – Alkalinity and PON on $\sigma_{26}$ for DIC+/-

Supplementary Figure 8 – Multiple linear regression model

References

| **Variable/Latitude** | **Bias** | **RMSD** | **Correlation** | **σ_model_** | **σ_observations_** |
| --- | --- | --- | --- | --- | --- |
| **Temperature** (°C)  42N  40N  38N  37N  35N | 0.6262  0.1558  0.3620  0.6467  0.3323 | 0.6669  1.007  0.8206  0.9049  1.296 | 0.9182  0.7314  0.8473  0.9048  0.8090 | 1.524  1.430  1.367  1.874  1.937 | 1.117  1.296  0.7764  1.269  0.9487 |
| **DIC** (mmol/$m^{3}$)  42N  40N  38N  37N  35N | -21.15  5.111  17.78  -32.91  7.822 | 42.50  35.83  27.53  30.67  46.56 | 0.8365  0.8246  0.8578  0.9010  0.7203 | 73.07  54.38  53.55  68.48  67.03 | 75.38  63.22  45.88  54.08  45.97 |
| **TAlk** (meq/$m^{3}$)  42N  40N  38N  37N  35N | 2.162  -2.545  -2.309  -9.689  -2.697 | 21.21  20.90  14.62  14.38  14.18 | 0.7063  0.5249  0.6548  0.5851  0.4795 | 19.21  16.98  15.98  17.13  13.40 | 29.85  24.02  18.70  13.74  14.36 |
| **pH** (total scale)  42N  40N  38N  37N  35N | 0.05674  -0.02096  -0.05755  0.07143  -0.02837 | 0.07629  0.05509  0.09503  0.06945  0.07569 | 0.8572  0.8787  0.6533  0.8566  0.8292 | 0.1476  0.1061  0.1046  0.1317  0.1342 | 0.1330  0.1149  0.1209  0.1271  0.1213 |

**Supplementary Table 1**. Summary of model-comparison statistics for the Taylor diagrams presented in Fig. 1.

| Property | Range of values | Sensitivity |
| --- | --- | --- |
| Temperature | 9.09 – 9.66 (°C*)* | 0.007641 |
| Salinity | 33.7 – 33.86 | -0.00471 |
| Dissolved inorganic carbon (DIC) | 2178 – 2198 (${mmolC/m}^{3}$*)* | -0.006954572 |
| Total alkalinity | 2246 – 2257 (${meq/m}^{3}$*)* | 0.007359559 |

**Supplementary Table 2.** Sensitivity analysis for aragonite saturation state. A sensitivity analysis following Turi et al., 2018, and using the CO2Sys_v2.1.xls was conducted to assess the sensitivity of $\Omega_{\mathrm{arag}}$ to temperature, salinity, dissolved inorganic carbon (DIC) and total alkalinity. $\Omega_{\mathrm{arag}}$ values were calculated by varying one property at a time while keeping the others constant. The range of values represents the upwelling season mean +/- upwelling season standard deviation along the bottom of the 100m isobath. Sensitivity is calculated as the slope of a linear regression between the property being varied and the resulting $\Omega_{\mathrm{arag}}$ values.

|  | **Intercept** | **DIC** | **Depth** $\boldsymbol{\sigma}_{\boldsymbol{26}}$ | $\boldsymbol{R}^{\boldsymbol{2}}$ | **AIC** | **DW p-value** | **SW p-value** | **BT p-value** |
| --- | --- | --- | --- | --- | --- | --- | --- | --- |
| 1 | ***1.4844 | 0.4082 |  | 0.09704 | 80.33986 | 0.7284 | 0.02675 | 0.02671 |
| 2 | ***1.4844 |  | ***0.8630 | 0.4336 | 69.61213 | 0.08425 | 0.3221 | 0.006822 |
| 4 | *** 1.4844 | **0.6434 | ***1.0124 | 0.6616 | 59.76403 | 0.1127 | 0.1916 | 0.8153 |

Signif. codes: 0 ‘***’ 0.001 ‘**’ 0.01 ‘*’ 0.05 ‘.’ 0.1 ‘ ’ 1

**Supplementary Table 3.** Summary statistics of linear regressions. Linear regressions were fit between log(severity) and two explanatory variables, the depth of $\sigma_{26}$ and the concentration of DIC at the depth of $\sigma_{26}$. Explanatory variables were fit independently and then together, and were scaled by their center values in order to compare coefficient. P-values from tests on the residuals are reported. The assumption of independence was verified using the Durbin-Watson (DW) test [1], the assumption of normality was verified using the Shapiro-Wilks (SW) test [1], and the assumption of constant variance was verified using the Bartlett test (BT) [3].

**Supplementary Figure 1.** Simulated (1988-2010) and observed (2017-2019) dissolved oxygen (left) and temperature (right) at nearshore moorings. Monthly climatological mean, standard deviation and minimum/maximum values are represented by box and whisker plots for observed (blue) and simulated (gray) values at (from top to bottom) Van Damme, Point Arena, Big Creek, and Point Buchon (see Fig. 2 for locations).


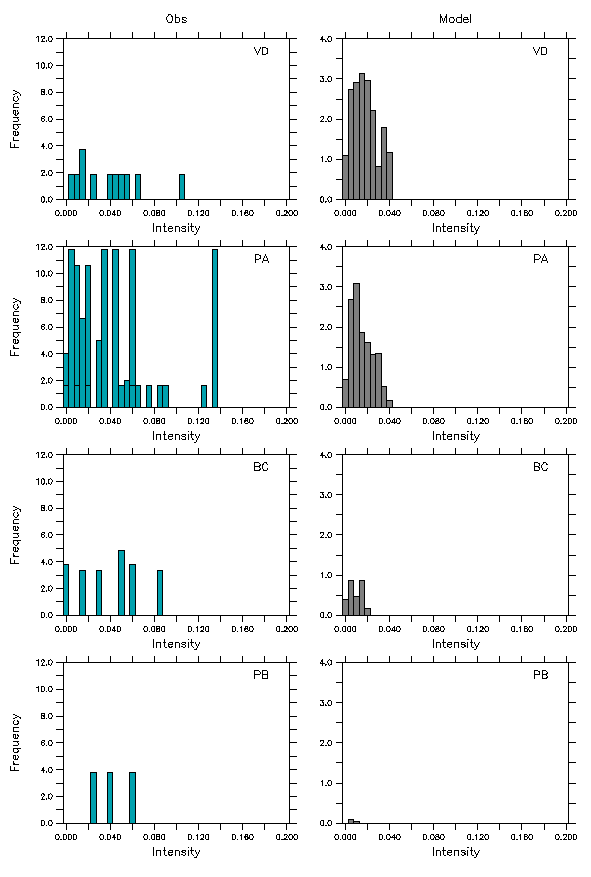

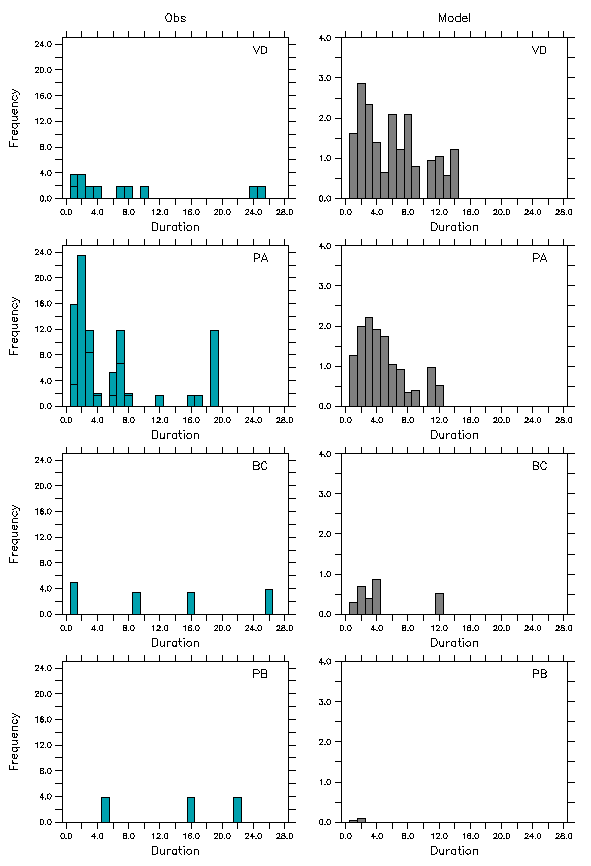


**Supplementary Figure 2.** Simulated (1988-2010) and observed (2017-2019) intensity and duration of low pH events (pH < 7.7). Frequency distribution of observed (blue) and simulated (gray) event duration (left panels) and intensity (right panels) at each nearshore mooring (VD=Van Damme, PA = Point Arena, BC = Big Creek, PB = Point Buchon; see Fig. 2 for locations). Event properties are normalized by the number of years of data.


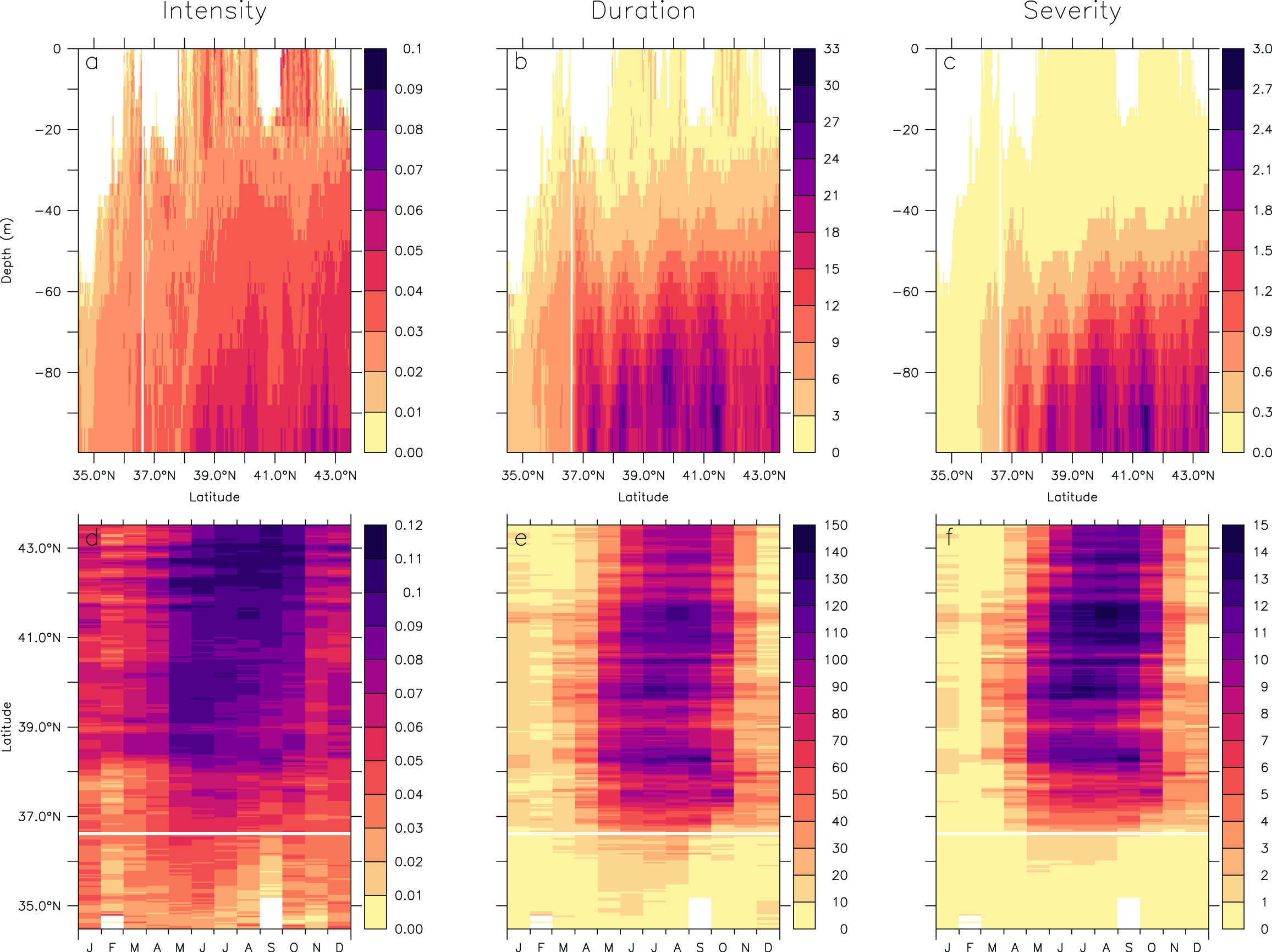


**Supplementary Figure 3.** Climatological properties of undersaturation events ($\Omega_{\mathrm{arag}}$ < 1). Top: event properties along the 100m isobath with respect to depth and latitude in terms of their monthly climatological intensity (a), duration in days (b), and severity (c). Bottom: Monthly climatology of event properties along the bottom of the 100m isobath with respect to latitude in terms of intensity (d), duration in days (e), and severity (f). White shading indicates absence of events.

**Supplementary Figure 4.** Fraction of the variability in $\Omega_{\mathrm{arag}}$ attributed to changes in DIC (red), total alkalinity (red), temperature (green) and salinity (blue) plotted as upwelling season averages as a function of latitude.


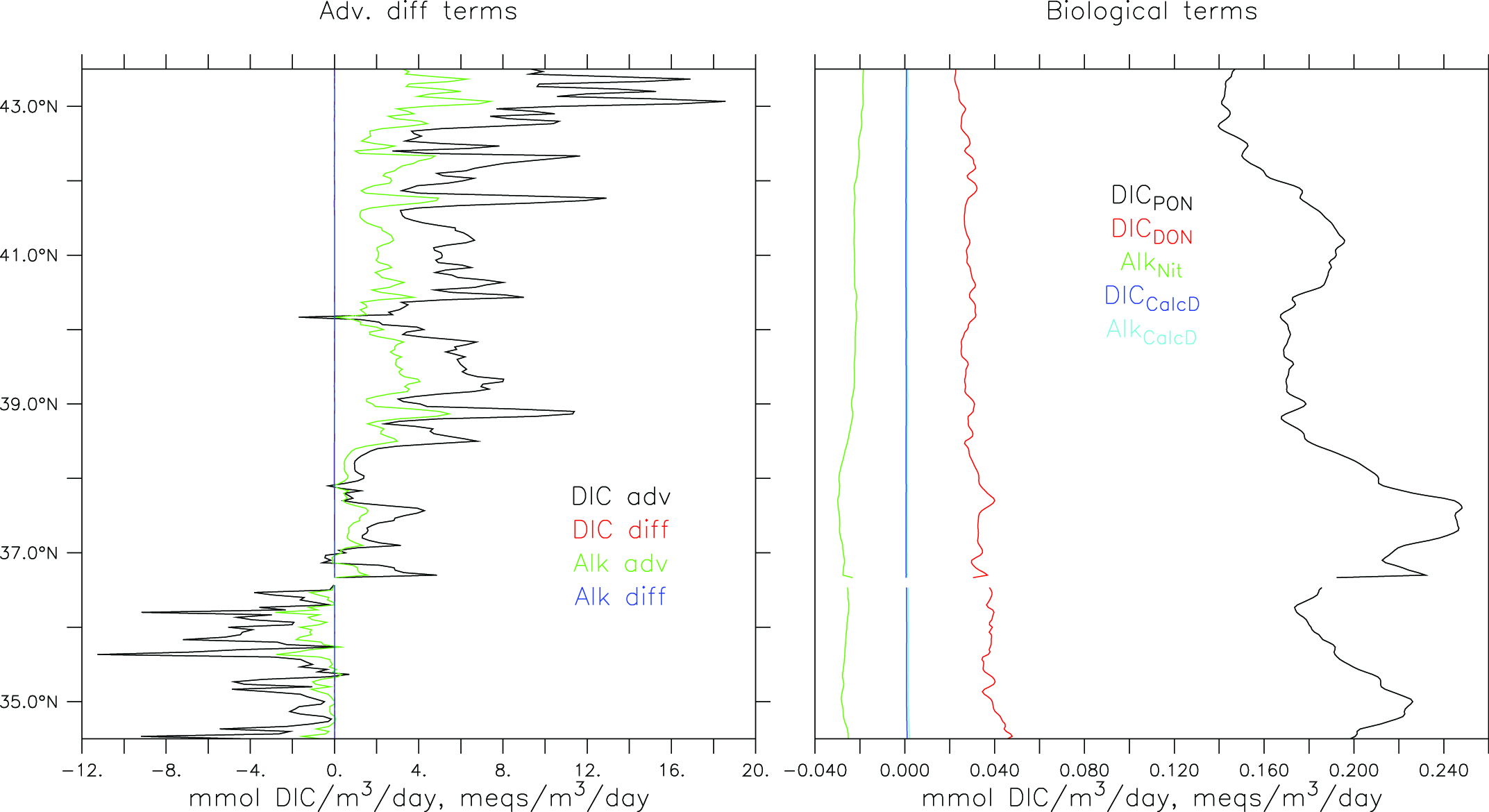


**Supplementary Figure 5.** Dissolved inorganic (DIC) and total alkalinity (TAlk) budget terms (mmol DIC/m^3^/day or meq/m^3^/day) along the bottom of the 100m isobath. (Left) Advection and mixing terms influencing DIC (black and red curves, repsetively) and Talk (green and blue curves, respectively) (note that the two mixing terms overlay each other as they are several orders of magnitude lower than advective terms). (Right) Biological processes influencing DIC and Talk, including remineralization of particulate organic nitrogen (PON) into ammonium (black), remineralization of dissolved organic nitrogen (DON) into ammonium (red), calcium carbonate dissolution (CalcD) (blue for DIC, cyan for TAlk), and nitrification (green). All values represent upwelling season means.


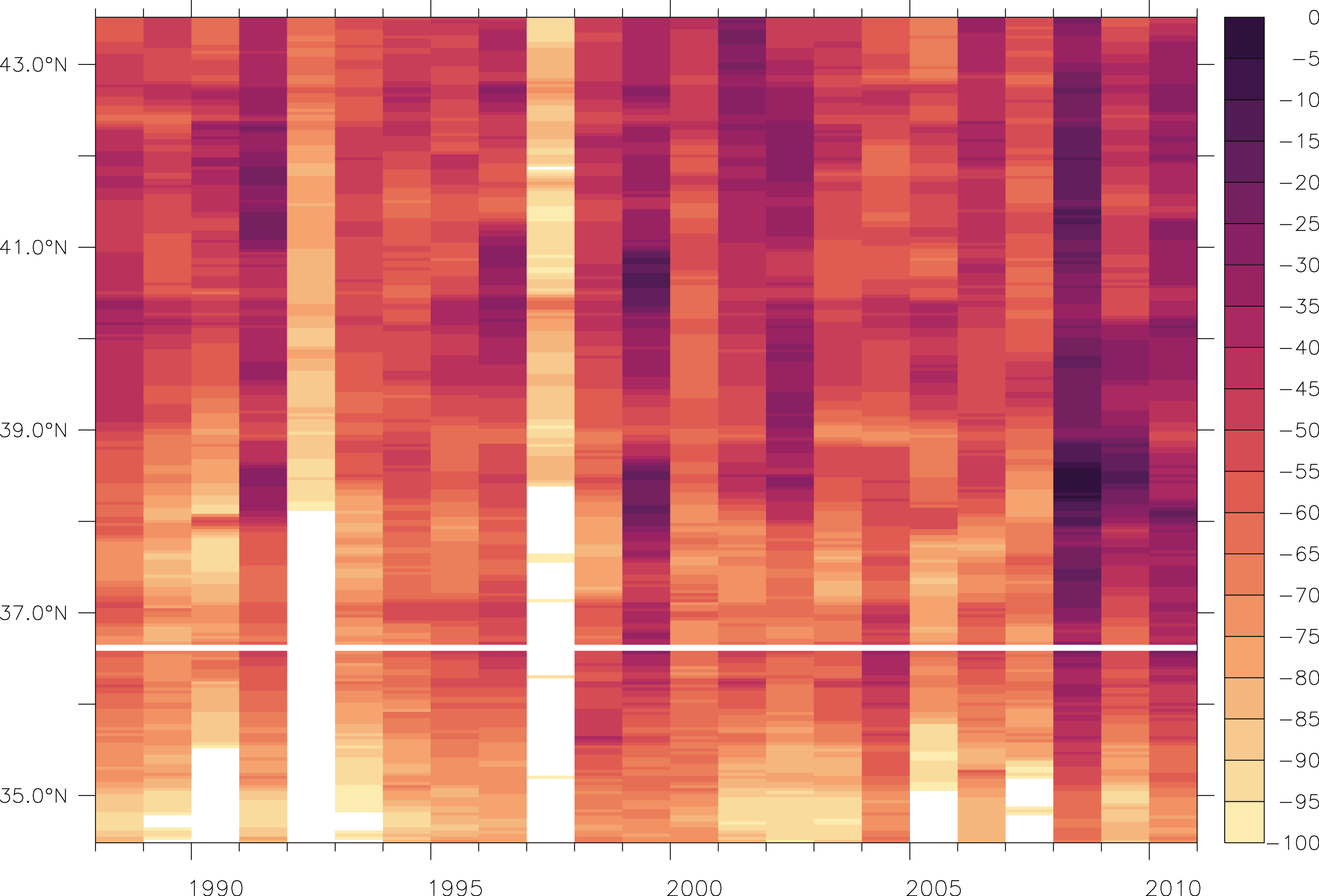


**Supplementary Figure 6.** Mean depth (m) of 26.0 kg/m^3^ isopycnal ($\sigma_{26}$ ) on the 100m isobath during the upwelling season (May-Sept.). Missing data indicate that $\sigma_{26}$ was deeper than 100m during the upwelling season.


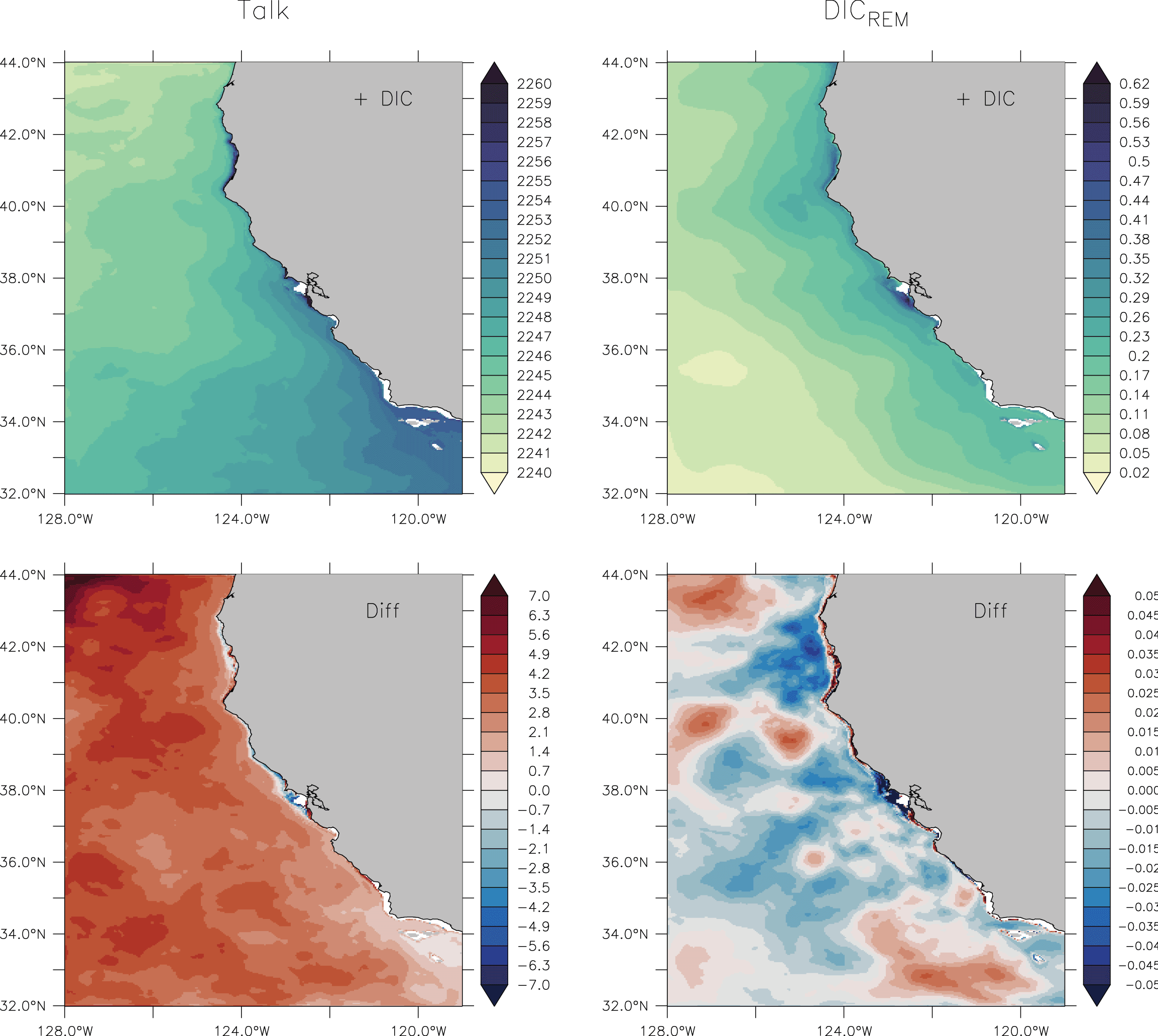


**Supplementary Figure 7.** Top: Upwelling-season mean alkalinity (meq/m^3^) (left) and $\mathrm{DIC}_{\mathrm{REM}}$ (mmolC/m^3^/day) (right). Bottom: Upwelling-season mean alkalinity (meq/m^3^) (left) and $\mathrm{DIC}_{\mathrm{REM}}$ (mmolC/m^3^/day) (right) differences between positive and negative phases of the first EOF mode for DIC (see Fig. 6).


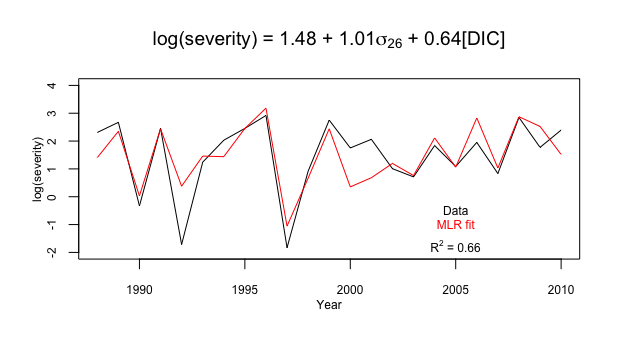


**Supplementary Figure 8.** Multiple linear regression model (red) with latitudinally averaged (35.5-43.5°N ) annual upwelling season (May-Sept) values of log severity (black) as a response variable and the scaled depth of 26 isopycnal ($\sigma_{26}$) and DIC concentrations at the depth of $\sigma_{26}$ averaged 0-200km offshore as explanatory variables. The MLR is given by log(severity) = 1.48 + 1.01($\sigma_{26}$) + 0.64([DIC]) and yields a multiple R-squared value of 0.66.

References:

1. Durbin, J. & Watson, G.S. Testing for serial correlation in least squares regression. *Biometrika* **37**, 409–428 (1950).
2. Royston, P. The W test for normality. *Applied Statistics*, **31**, 176–180 (1982).
3. Bartlett, M. S. Properties of sufficiency and statistical tests. *Proceedings of the Royal Society of London Series A* **160**, 268–282 (1937).
